# Large Propulsion Demands Increase Locomotor Adaptation at the Expense of Step Length Symmetry

**DOI:** 10.3389/fphys.2019.00060

**Published:** 2019-02-08

**Authors:** Carly J. Sombric, Jonathan S. Calvert, Gelsy Torres-Oviedo

**Affiliations:** Sensorimotor Learning Laboratory, Department of Bioengineering, University of Pittsburgh, Pittsburgh, PA, United States

**Keywords:** human, split-belt locomotion, motor adaptation, kinetics, kinematic, gait, walking

## Abstract

There is an interest to identify factors facilitating locomotor adaptation induced by split-belt walking (i.e., legs moving at different speeds) because of its clinical potential. We hypothesized that augmenting braking forces, rather than propulsion forces, experienced at the feet would increase locomotor adaptation during and after split-belt walking. To test this, forces were modulated during split-belt walking with distinct slopes: incline (larger propulsion than braking), decline (larger braking than propulsion), and flat (similar propulsion and braking). Step length asymmetry was compared between groups because it is a clinically relevant measure robustly adapted on split-belt treadmills. Unexpectedly, the group with larger propulsion demands (i.e., the incline group) changed their gait the most during adaptation, reached their final adapted state more quickly, and had larger after-effects when the split-belt perturbation was removed. We also found that subjects who experienced larger disruptions of propulsion forces in early adaptation exhibited greater after-effects, which further highlights the catalytic role of propulsion forces on locomotor adaptation. The relevance of mechanical demands on shaping our movements was also indicated by the steady state split-belt behavior, during which each group recovered their baseline leg orientation to meet leg-specific force demands at the expense of step length symmetry. Notably, the flat group was nearly symmetric, whereas the incline and decline group overshot and undershot step length symmetry, respectively. Taken together, our results indicate that forces propelling the body facilitate gait changes during and after split-belt walking. Therefore, the particular propulsion demands to walk on a split-belt treadmill might explain the gait symmetry improvements in hemiparetic gait following split-belt training.

## Introduction

There is an interest in increasing the extent of locomotor adaptation induced by split-belt walking because repeated exposure to this task can lead to gait improvements post-stroke. Notably, promising studies have shown that walking with the legs moving at different speeds (i.e., split-belt walking) results in long-lasting reduction of step length asymmetry post-stroke when walking overground (Reisman et al., 2013; Betschart et al., 2018; Lewek et al., 2018). This is important for gait rehabilitation post-stroke because step length asymmetry can lead to other comorbidities such as musculoskeletal injuries (Jørgensen et al., 2000) and joint pain (Patterson et al., 2012). While split-belt walking could be beneficial, it is not effective in all stroke individuals (Reisman et al., 2013; Lewek et al., 2018) and it is still unclear why some stroke survivors re-learn to walk symmetrically but others do not. Thus, it is fundamental to identify factors contributing to split-belt adaptation so that we can manipulate them to facilitate motor corrections in all individuals.

The increased mechanical work (Selgrade et al., 2017), step length asymmetry (Reisman et al., 2005), and hence metabolic effort (Finley et al., 2013), upon introducing the split-belt environment are thought to drive locomotor adaptation. Notably, these three factors are large during the initial steps of split-belt walking and are minimized as subjects learn to walk in the split-belt context (Finley et al., 2013; Selgrade et al., 2017). Thus, modulating anterior–posterior forces applied at the feet during split-belt walking could facilitate locomotor adaptation given their direct impact on mechanical work and step lengths in regular gait (Donelan et al., 2002). In particular, we hypothesized that altering braking forces could modulate the adaptation of gait based on prior studies showing that braking forces are tightly regulated during split-belt walking (Ogawa et al., 2014). Notably, braking forces are suddenly disrupted when the split-belt perturbation is introduced and this perturbation is reduced as subjects adapt their gait. Thus, we proposed that braking forces could facilitate subject-specific locomotor adaptation and therefore, increasing these forces would augment the extent of gait changes during and after this task (i.e., after-effects).

To test this hypothesis, we assessed subjects’ gait before, during, and after split-belt walking at different inclinations, which naturally and distinctively modulated the braking and propulsion forces experienced at the feet (Lay et al., 2006, 2007). We found that inclination increased locomotor adaptation and after-effects as hypothesized, but not through the proposed mechanism. While the braking and propulsion forces were adapted and exhibited after-effects, it was the propulsion forces that augmented the gait changes during and after split-belt walking. Specifically, decline walking, which accentuated braking forces, did not lead to as much adaptation of step length asymmetry as incline walking, which augmented propulsion forces. In addition, propulsion forces during baseline and early adaptation were highly predictive of step length after-effects at an individual level. Interestingly, each inclination group recovered their baseline leg orientation to meet force demands at the expense of step length symmetry, which was surprising given the consistent human tendency to self-select symmetric step lengths in the split-belt environment (Yokoyama et al., 2018). Taken together, our findings suggest that propulsion demands, rather than braking ones, facilitate locomotor adaptation and after-effects induced by split-belt walking.

## Materials and Methods

We investigated the effect of modulating anterior–posterior forces applied at the feet on gait adaptation during and after split-belt walking under distinct slopes (i.e., flat, decline, and incline), which naturally altered braking and propulsion forces (Lay et al., 2006, 2007). To this end, we evaluated the kinetic and kinematic adaptation and after-effects of 24 young healthy subjects (12 men and 12 women, 24.5 ± 4.9 years of age) randomly assigned to one of three groups experiencing the split-belt adaptation protocol in a flat, incline, or decline configuration (*n* = 8 each). Written and informed consent was obtained from all participants prior to participation. The University of Pittsburgh Institutional Review Board approved the experimental protocol, which conformed to the standards set by the Declaration of Helsinki, except for registration in a database.

### General Paradigm

**FIGURE 1:**
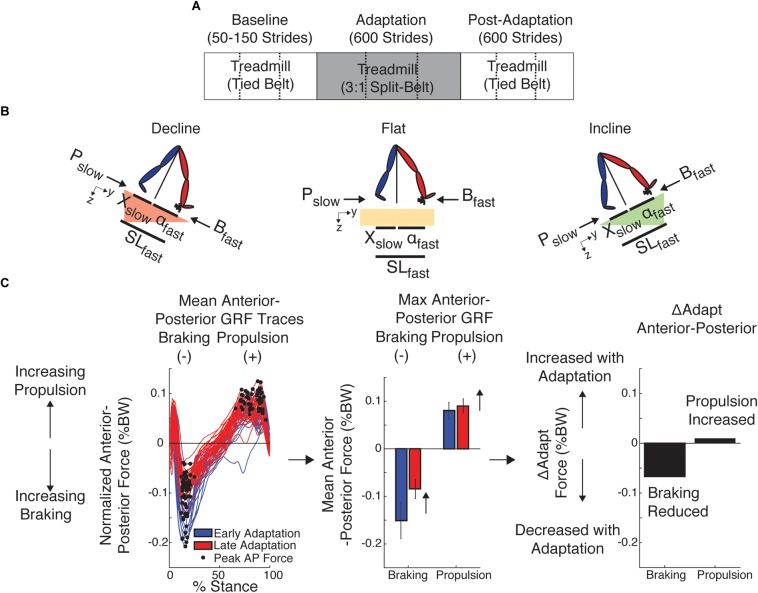
Experimental Paradigm and Kinetic and Kinematic Analysis. **(A)** Paradigm used for all sloped conditions to assess locomotor adaptation during and after split-belt walking. Subjects from each group walked either incline (8.5°), flat, or decline (–8.5°) throughout the experiment, including baseline and post-adaptation. All groups experienced the same number of strides per epoch (baseline: between 50 and 150, adaptation: 600, post-adaptation: 600). Dashed lines indicate when the resting breaks occurred. **(B)** The decomposition of step length into leading (α) and trailing (*X*) leg positions with respect to the body is illustrated for each sloped condition. This decomposition was done because it is known that inclination affects these aspects of step length differently (Leroux et al., 2002; Dewolf et al., 2017, 2018). Also note that when taking a step, the step length will depend on the position of the leading and trailing leg, which are generating a braking and propulsion force, respectively. **(C)** We used the peak braking and peak propulsion force for each step to compute outcome measures of interest, such as the ΔAdapt measure. This measure was computed to quantify increments or reductions in magnitude within the adaptation epoch of each specific parameter. Note that increases in magnitude were defined as positive changes, whereas decreases in magnitude were defined as negative changes.

All subjects experienced the same split-belt paradigm illustrated in Figure 1A. Only the inclination at which subjects walked was altered across groups to determine the role of anterior–posterior ground reaction forces on the adaptation and after-effects of kinetic and kinematic gait features. We specifically tested subjects at three inclinations: flat (0°), incline (8.5°), or decline (-8.5°). These slopes were selected based on previous studies investigating walking on inclined surfaces (e.g., Lay et al., 2006) and because greater inclinations were too strenuous for split-belt walking, as assessed in pilot studies. Subjects from each group walked at the specified inclination throughout the experiment, including baseline and post-adaptation. Baseline: Subjects first experienced a baseline epoch to characterize their gait at the specific inclination at which they were going to walk throughout the study. During the baseline epoch, subjects walked with the belts moving at the same speeds (i.e., tied condition). Belts moved either at a slow (0.5 m/s), fast (1.5 m/s), and medium (1 m/s) speeds for at least 50 strides. Strides were counted in real-time using raw kinetic data. A stride was defined as the period between two consecutive heel strikes (i.e., foot landing) of the same leg. Adaptation: The adaptation epoch was used to assess subjects’ ability to adjust their locomotor pattern in response to a split-belt perturbation. During this period, one leg moved three times faster than the other (0.5 and 1.5 m/s) for 600 strides (∼10 min), except for one subject in the decline group who experienced the adaptation condition for 907 strides (due to technical difficulties in heel strike detection). This subject was not excluded from the analysis given that this participant’s behavior did not differ from that of other subjects and its inclusion or exclusion did not alter our conclusions. The leg walking fast was the dominant leg, which was determined as the self-reported leg used to kick a ball. Post-adaptation: This epoch was used to assess the after-effects when the split-belt condition was removed. Subjects experienced tied walking at the medium speed (1 m/s) for 600 strides at their group-specific slope. The speeds for the adaptation and post-adaptation periods were chosen based on other studies in young subjects (e.g., Reisman et al., 2005) so that the fast speed in the 3:1 belt ratio was not at the walk-to-run speed transition for any participant. The duration of the adaptation and post-adaptation period was comparable to studies in healthy and clinical populations (e.g., Reisman et al., 2009) to ensure that all individuals had enough exposure to the split condition and could complete the entire protocol at all inclinations. In addition, subjects in all groups where given resting breaks every 200 strides during the adaptation and post-adaptation epochs to prevent fatigue.

### Data Collection

Kinematic and kinetic data were used to characterize subjects’ ability to adapt their gait during adaptation, and maintain the learned motor pattern during post-adaptation. Kinematic Data: Kinematic data were collected with a passive motion analysis system at 100 Hz (Vicon Motion Systems, Oxford, United Kingdom). Subjects’ behavior was characterized with passive reflective markers placed symmetrically on the ankles (i.e., lateral malleolus) and hips (i.e., greater trochanter) and asymmetrically on shanks and thighs (to differentiate the legs). The origin of the kinematic data was rotated with the treadmill in the incline and decline conditions such that the *z*-axis (‘vertical’ in the flat condition) was always orthogonal to the surface of the treadmill (Figure 1B). Gaps in raw kinematic data were filled with a quintic spline interpolation (Woltring; Vicon Nexus Software, Oxford, United Kingdom). Kinetic Data: Kinetic data were collected with an instrumented split-belt treadmill at 1,000 Hz (Bertec, Columbus, OH, United States). Force plates were zeroed prior to each testing session so that each force plate’s weight did not affect the kinetic measurements. In addition, the reference frame was rotated at the inclination of each specific experiment such that the anterior–posterior forces were aligned with the surface on which the subject walked. A heel strike was identified in real time when the raw normal force under each foot reached a threshold of 30 Newtons. This threshold was chosen to ensure accurate identification of foot landing at all sloped conditions. On the other hand, we used a threshold of 10 Newtons on median filtered data (with a 5 ms window) to detect the timing of heel strikes more precisely for data processing.

### Data Analysis

#### Kinematic Data Analysis

Kinematic behavior was characterized with step length asymmetry, which exhibits robust adaptation in split-belt paradigms (e.g., Reisman et al., 2005). It is calculated as the difference in step length between the two legs on consecutive steps. Step length (SL) is defined as the distance in millimeters between the ankle markers at heel strike. Therefore, equal step lengths result in zero step length asymmetry, whereas different step lengths result in non-zero step length asymmetry. A positive step length asymmetry indicates that the fast leg’s step length (which for this study is the dominant leg’s step length) is longer than the slow leg’s step length. Step length asymmetry was normalized by stride length, which is the sum of two consecutive step lengths, resulting in a unitless parameter that is robust to inter-subject differences in step size.

Each step length was also decomposed into anterior and posterior components relative to the hip position (Figure 1B) as in previous work (Finley et al., 2015). This was done to characterize the leading and trailing legs’ orientation relative to the body when taking a step given that inclination is known to affect these measures (Leroux et al., 2002; Dewolf et al., 2017). The leading leg’s orientation was characterized by the anterior component, ‘α’, which we computed as the distance in millimeters between the leading leg’s ankle and the hip at heel strike; similarly, the trailing leg’s orientation was characterized by the posterior component, ‘*X*,’ which we computed as the distance in millimeters between the trailing leg’s ankle and the hip at heel strike. The hip position, which is a proxy for the body’s position, was estimated as the mean instantaneous position across hip markers. By convention positive α values indicated that the foot landed in front of the hips, whereas negative *X* values indicated that the trailing leg was behind the hips at foot landing. Note that α and *X* when taking a step not only indicated the leg orientation of the leading and trailing legs, respectively, but their magnitudes sum to the leading leg’s step length. As indicated in Figure 1B, α and *X* were computed aligned to the treadmill‘s surface in all sloped conditions.

#### Kinetic Data Analysis

Kinetic data were used to characterize the adaptation of ground reaction forces. The kinetic analysis was focused on forces in the anterior–posterior direction since these are modulated by inclination (e.g., Lay et al., 2006) and they are adapted during split-belt walking (Ogawa et al., 2014). The anterior–posterior ground reaction forces (AP forces) were first low-pass filtered with a cutoff frequency of 20 Hz. Then, they were normalized by each subject’s body weight to account for inter-subject differences.

The AP forces were further decomposed into peak braking and peak propulsion forces for each stride. The peak braking force was quantified as the minimum value of the AP force for the slow and fast leg (Figure 1C; *B*_slow_ and *B*_fast_, respectively), whereas the peak propulsion force was quantified as the maximum value of the AP force for the slow and fast leg (Figure 1C; *P*_slow_ and *P*_fast_, respectively). We systematically excluded maxima values occurring before the braking force. Thus, we did not consider the initial positive AP forces following heel strike in the identification of propulsion forces. Peak forces were used to characterize braking and propulsion to be consistent with prior split-belt studies (Mawase et al., 2013; Ogawa et al., 2014) and those reporting kinetic differences between inclinations (Lay et al., 2006; Item-glatthorn et al., 2016). Note that we did not remove slope-specific biases due to gravity because we focused on analyzing changes in braking and propulsion forces between epochs of interest.

#### Kinetic and Kinematic Outcome Measures

Outcome measures were used to characterize the adaptation and after-effects of kinematic and kinetic gait features in response to a split-belt perturbation. A comprehensive list of outcome measures is provided in Table 1. Medium baseline behavior was used as a reference in all outcome measures computed with kinematic parameters (e.g., step length asymmetry and step lengths), whereas speed-specific baselines were used for kinetic parameters (e.g., braking and propulsion forces). In other words, fast baseline was used as a reference for the leg walking fast during adaptation and the slow baseline was used as a reference for the leg walking slow during adaptation, whereas medium baseline was used as a reference for both legs when they walked at the same medium speed in post-adaptation. This methodology is consistent with prior split-belt studies indicating that kinetic parameters plateau near values similar to those of the speed-specific baseline (Ogawa et al., 2014). Outcome measures of interest were Early Adaptation, Late Adaptation, After-Effects, ΔAdapt, and ΔPost. Early Adaptation (EarlyA) was defined as the difference between the averaged behavior of the first 5 strides of the split-belt condition (strides 1–5) and the speed-specific baseline values as indicated earlier. This outcome measure characterized the extent to which subjects were perturbed by the split-belt condition. Note that we did not exclude any strides of adaptation because all subjects experienced a short split-belt condition before the adaptation epoch to minimize startle effects. Late Adaptation was defined as the average of the last 40 strides of adaptation for all parameters. This outcome measure indicated the steady state behavior reached at the end of the adaptation epoch. After-Effects were defined as the average of the first 5 strides of Post-Adaptation relative to medium baseline such that increments and reductions in magnitude of a specific parameter with respect to medium baseline were marked as positive or negative, respectively. We also characterized the behavioral changes within adaptation and post-adaptation with indices ΔAdapt and ΔPost, respectively. ΔAdapt and ΔPost were computed as the difference between Late and Early Adaptation for ΔAdapt and late and early Post-Adaptation for ΔPost (i.e., average of the last 40 strides or 5 strides for late and early, respectively). This was done such that an increase in the magnitude of a parameter during either adaptation or post-adaptation resulted in positive values and a reduction of the parameter was marked as negative values. For example, we illustrate ΔAdapt for braking and propulsion forces in Figure 1C. Note that the braking force decreased during the adaptation epoch (negative value), whereas the propulsion force increased during the same epoch (positive value). Lastly, the rate of adaptation was determined by fitting the averaged step length asymmetry for each inclination group with a single exponential [*y* = *a*^∗^exp((-1/τ)^∗^*x*)+*c*] using a non-linear least squares method.

**Table 1:** Outcome measures.

Outcome measure	Meaning
Early Adaptation (EarlyA)	Quantifies the extent to which any parameter is disrupted by split-belt walking. The bias of this outcome measure is removed using either medium baseline or speed-specific baseline for symmetry parameters and leg-specific parameters, respectively.
Late Adaptation	Quantifies the steady state value for step length asymmetry during the split-belt adaptation epoch. We removed the bias of this measure during medium baseline.
After-Effects	Quantifies short-term gait changes following split-belt walking relative to medium baseline. Positive values indicate that post-adaptation values are larger in magnitude than it was during baseline.
ΔAdapt and ΔPost	Quantifies the change in a parameter during adaptation and post-adaptation, respectively. Therefore, ΔAdapt and ΔPost enabled us to determine if changes during adaptation led to comparable changes during post-adaptation. Positive values mean that there is an increase in magnitude of a parameter within an epoch and vice versa.
Rate of Adaptation	Quantifies the group’s adaptation rate of step length asymmetry when using a single exponential to fit step length asymmetry during the split-belt adaptation epoch.


### Statistical Analysis

#### Planned Analysis

Statistical analyses were used to determine the effect of slope on group behavior. In particular, we consider that the distinct sloped conditions could either alter the magnitude of adaptation, after-effects and/or the rate at which subjects adapted. Thus, we tested the effect of slope on each of these aspects separately. More specifically, we performed separate one-way ANOVAs to test the effect of sloped condition on each kinetic and kinematic outcome measure quantifying the magnitude of subjects’ adaptation and after-effects (e.g., ΔAdapt, After-Effects, etc.). Outcome measures with significant group main effects were further analyzed with Tukey *post hoc* testing. On the other hand, to test the effect of slope on adaptation rate, we compared the 95% confidence intervals for the τ quantifying each group’s adaptation rate. Time constants were determined to be significantly different when the confidence intervals were not overlapping. This was done, as opposed to ANOVAs because single fits of group data were more representative of the group data (fit of incline group: *R*^2^ = 0.86; flat group: R^2^ = 0.94; decline group R^2^ = 0.95) than exponential fits of individual subjects. We additionally wished to know if each group’s step length asymmetry steady state was different from zero, therefore we performed a two-sided *t*-tests on each group’s late adaptation values. A three-way ANOVA was also performed to determine the effect of leg and inclination condition on the changes of step length that occurred during adaptation and post-adaptation. As such, the dependent variables were changes of step length that occurred during these epochs (i.e., ΔAdapt and ΔPost); the independent variables were slope (i.e., incline, flat, decline), leg (i.e., fast or slow leg), epoch (i.e., adaptation or post-adaptation), and the interactions between these independent variables. A significance level of α = 0.05 was used for all statistical tests. All statistical analyses were performed with MATLAB (The MathWorks, Inc., Natick, MA, United States).

#### *Post hoc* Analysis

Following our planned analyses, we realized that (1) each group reached distinct step length asymmetry values during late adaptation and that (2) the incline group, which had the largest propulsion demands had the largest step length asymmetry after-effects. Thus, we performed distinct regression analyses to further investigate these two findings.

First, to investigate the distinct late adaptation values, we quantified the similarity between leg orientations across baseline and late adaptation epochs with a linear regression analysis. We specifically tested the model *y* = *a*^∗^*x*, where *y* is the predicted leg orientation during late adaptation and *x* is the leg orientation during baseline. In the same vain, we performed a regression analysis between leg orientations across late adaptation and early post-adaptation. However, in this latter case the trailing legs’ orientation was hypothesized to be contralaterally correlated across epochs (i.e., the trailing leg orientation of one leg during early post-adaptation was regressed against the trailing leg orientation of the other leg during late adaptation).

Second, we used linear models to test the potential relevance of each leg’s propulsion and braking forces on step length asymmetry after-effects. Specifically, we regressed a categorical group factor and each leg’s braking or each leg’s propulsion forced during early adaptation against step length asymmetry after-effects (i.e., at total of 4 multiple regressions). We subsequently performed linear regressions between kinetic and kinematic variables within and across experimental epochs to understand the exclusive relation between altering the slow leg’s Early Adaptation propulsion force and step length asymmetry After-Effects. Of note, the regression of propulsion forces across epochs (i.e., between ΔAdapt and After-Effects) was done with contralateral legs to be consistent with the *post hoc* observation that adaptation of step length on one side led to after-effects on the other side (see Figure 3). In these regressions, group was used as a categorical factor only if it was found to be independent of the continuous variable as indicated by a non-significant Pearson Coefficient. If a strong correlation between the categorical and continuous regressor was found, a linear regression was performed and the confounding influence of group was noted. For visualization purposes only, we displayed the results of a linear regression when the continuous variable of the linear model was a significant factor.

## Results

### Inclination Regulated the Adaptation and After-Effects of Step Length Asymmetry

**FIGURE 2:**
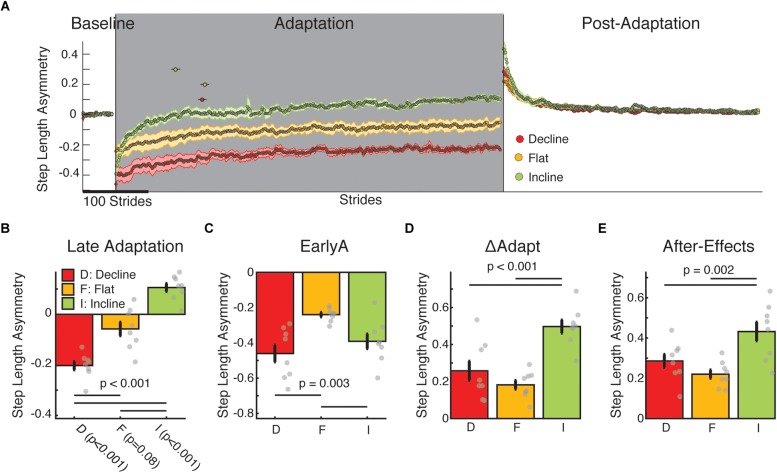
Step length asymmetry adaptation and after-effects. **(A)** Stride-by-stride time course of step length asymmetry during medium baseline, adaptation, and post-adaptation are shown. Each data point represents the average of 5 consecutive strides and shaded regions indicate the standard error for each group. The dots above the time courses indicate the time constants with 95% confidence intervals. Note that the incline group adapted more quickly than the other sloped conditions as indicated by the smaller time constant (τ) and not overlapping 95% confidence interval for this group compared to the other two groups [incline: 132.0 strides (125.6–138.4); flat: 135.6 strides (128.8–142.4); decline: 91.3 strides (84.7–97.9)]. These time constants are reflective of the data given that the exponential fits were able to characterize the data well (incline: *R*^2^ = 0.86; flat: *R*^2^ = 0.94; decline: *R*^2^ = 0.95). **(B–E)** We display group average values for outcome measures using step length asymmetry ± standard errors. Thin horizontal lines between groups illustrate significant differences (*p* < 0.05) based on *post hoc* analysis and gray dots indicate values for individual subjects. **(B)** Late adaptation: The height of the bars indicates group average step length asymmetry during late adaptation ± standard errors. Note that each group plateaued at different step length asymmetry values and the incline and decline groups had steady states statistically different from symmetry. Individual *t*-tests are reported on the *x*-axis. **(C)** EarlyA: The height of the bars indicates group average step length asymmetry during early adaptation ± standard errors. The sloped groups (both incline and decline) were more perturbed than the flat group by the split-belt perturbation. **(D)** ΔAdapt: The height of the bars indicates group average change of step length during the adaptation epoch ± standard errors. The incline group modulates their step length asymmetry more during adaptation than the flat or decline group. **(E)** After-effects: The height of the bars indicates group average step length after-effects early in the post-adaptation epoch ± standard errors. Again, the incline group had greater locomotor after-effects than the flat or decline groups.

**FIGURE 3:**
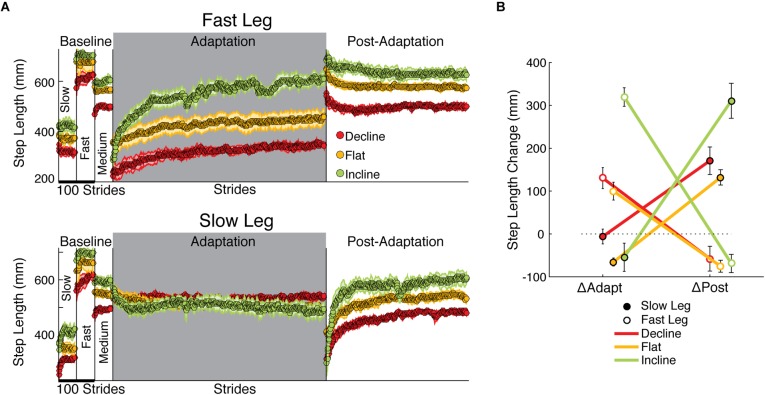
Step length adaptation and after-effects. **(A)** Stride-by-stride time courses of step lengths when either the fast leg (top panel) or the slow leg is leading (bottom panel) are shown during slow baseline, fast baseline, medium baseline, adaptation, and post-adaptation. Each data point represents the average of 5 consecutive strides and shaded regions indicate the standard error for each group. Step lengths were different across groups in all baseline speeds. In addition, the fast step length of the incline group exhibited the largest change during the adaptation epoch (largest ΔAdapt across groups). In contrast, the slow step length was similarly adapted across groups and approached the same value at steady state in all inclination conditions (*p* = 0.17 in one-way ANOVA of biased values during Late Adaptation). On the other hand, the slow step length had large and slope-mediated after-effects, whereas the fast step length had modest and similar after-effects across sloped conditions. **(B)** The effect of slope on each leg’s change during adaptation (ΔAdapt) and post-adaptation (ΔPost) is illustrated. Note the contralateral relation between adaptation and post-adaptation in all sloped conditions: the fast step length adapted more than the slow one (large ΔAdapt for fast leg), whereas the slow step length had most of the after-effects (large ΔPost for slow leg). This contralateral relation is exaggerated by incline walking.

**FIGURE 4:**
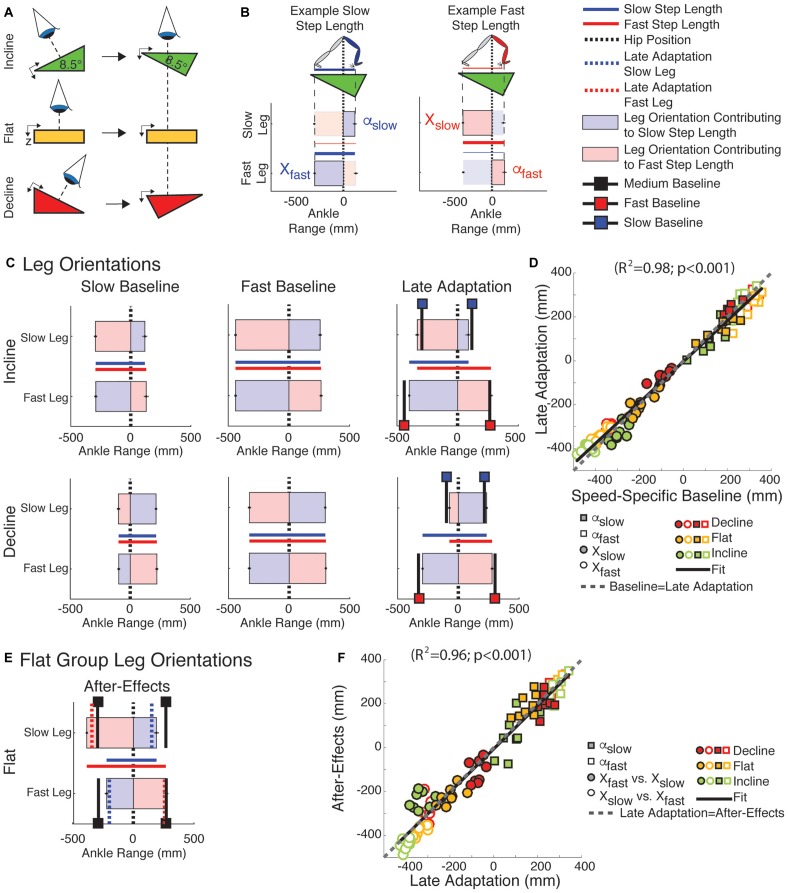
Leg orientation adaptation and after-effects. **(A)** Schematic indicating the view projected in **(B–F)**. **(B)** Visualization used in **(C,E)**. Vertical zero lines represent the perpendicular projection of the hips onto the treadmill. The horizontal bars represent the ankle positions with respect to the hips at ipsilateral and contralateral heel strikes ± standard errors. The leading and trailing leg’s orientation (α and *X*, respectively) at heel strike are presented when taking a step with either the slow or fast leg. The horizontal lines plotted between the two horizontal bars represent the step lengths for when the fast leg is leading (in blue) or for when the slow leg is leading (in red). **(C)** Leg orientations are illustrated for both legs during slow baseline (1st column), fast baseline (2nd column), and late adaptation (3rd column). Black lines indicate baseline leg orientations at slow (black lines with blue squares) or fast (black lines with red squares) speeds. Leg orientations were similar between speed-specific baseline and late adaptation resulting in asymmetric step lengths in the sloped conditions. **(D)** The similarity between leg orientations across speed-specific baseline and late adaptation epochs is illustrated by the significant regression (*y* = *a*^∗^*x*, 95% Confidence interval for *a* = [0.91, 0.96]). Note that the regression line closely overlaps with the idealized situation in which baseline and late adaptation values are identical (dashed gray line; slope of one, i.e., *y* = *x*). **(E)** Leg orientations are illustrated during early post-adaptation (After-Effects) in the flat group. We also plotted the α and *X* during medium baseline (black lines with black squares) and during late adaptation (red and blue dotted lines). Note the ipsilateral similarity between the leading leg’s orientation (α) across late adaptation and early post-adaptation contrasting the contralateral similarity between trailing leg’s orientation across these two epochs. **(F)** The ipsilateral and contralateral similarity between α and *X*, respectively, across the late adaptation and early post-adaptation epochs is quantified with a signification correlation (*y* = *a*^∗^*x*, 95% Confidence interval for *a* = [0.94, 1.03]). The idealized situation in which late adaptation and early post-adaptation values are identical is presented as a reference (dashed gray line; slope of one, i.e., *y* = *x*).

Step length symmetry was not recovered in the sloped split-belt conditions. All groups were perturbed by split-belt walking and subsequently adapted (Figure 2A). However, each group reached a different step length asymmetry by Late Adaptation (*p* < 0.001) such that the flat group plateaued near values that were not different from zero (*p* = 0.08) (i.e., symmetric step lengths), whereas the decline group undershot step length symmetry (*p* < 0.001) and the incline group overshot step length symmetry (*p* < 0.001) (Figure 2B). Interestingly, while both sloped groups were perturbed more than the flat group (Figure 2C; EarlyA: group main effect *p* = 0.003; incline vs. flat *p* = 0.036; decline vs. flat *p* = 0.002), only the incline group adapted more than the other two groups (Figure 2D; ΔAdapt: group main effects *p* < 0.001; incline vs. flat *p* < 0.001; incline vs. decline *p* = 0.002) and faster (Figure 2A; dots above the adaptation epoch time courses indicate the time constants with 95% confidence intervals). Importantly, groups were not only distinct in their adaptation, but also in their after-effects (Figure 2E; *p* = 0.002) such that the incline group had greater after-effect than the decline (*p* = 0.03) and flat groups (*p* = 0.002). Therefore, contrary to our hypothesis, the slope condition augmenting propulsion (i.e., incline walking) rather than braking led to more and faster adaptation and greater after-effects. It was also unexpectedly found that the sloped groups plateaued at values that were distinct from their baseline step length symmetry, begging the questions of whether inclination had a different effect across individual step lengths (addressed in Figure 3) and why this would happen (addressed in Figure 4).

### Inclination Accentuates the Adaptation of Step Lengths on the Fast Leg and Subsequent After-Effects on the Slow Leg

While incline walking augmented the adaptation and after-effects of step length asymmetry, it had a unilateral effect on individual step lengths: it predominantly increased the adaptation of one leg and the after-effects of the other leg. Figure 3 illustrates the time courses for each step length at each slope. While both legs adapted and had after-effects in all groups, the adaptation of the fast leg was greater than that of the slow leg, whereas the opposite was observed during post-adaptation. In other words, the leg that was modified the most switched between the fast and slow legs depending on the epoch, which was substantiated by the significant interaction between leg and epoch on the changes of step length (Figure 3B; leg#epoch *p* < 0.001). In addition, the sloped conditions augmented this switching between legs, as indicated by the significant interaction between leg, epoch, and slope (leg#epoch#slope *p* < 0.001). Once more, the incline group drove this effect by exhibiting the largest changes on the fast side during adaptation (ΔAdapt: incline vs. flat *p* < 0.001; ΔAdapt: incline vs. decline *p* < 0.001; ΔAdapt: flat vs. decline *p* = 0.65) and on the slow side during post-adaptation (ΔPost: incline vs. flat *p* = 0.003; ΔPost: incline vs. decline *p* = 0.02; ΔPost: flat vs. decline *p* = 0.70). The impact of inclination on the changes of step length was further substantiated by the significant effect of slope (*p* < 0.001) in contrast with the non-significant effect of leg (*p* = 0.13) or epoch (*p* = 0.89). In sum, incline walking exaggerated the split-belt phenomenon of predominantly adapting the step length of the fast leg and observing after-effects on the other leg during post-adaptation.

### Slope- and Speed-Specific Walking Demands Determine the Distinct Step Length Asymmetry Across Inclination Conditions

Leg orientation mediated by inclination and walking speed caused the distinct step length asymmetries across sloped conditions. More specifically, subjects oriented each leg to prioritize speed- and slope-specific demands on AP forces at the expense of step length symmetry during adaptation. This is shown in Figure 4, which illustrates the top-down view of ankle positions relative to the body (Figure 4A) and the step lengths to which these positions contribute (Figure 4B) at baseline walking (slow and fast speeds) and late adaptation under each sloped condition (Figure 4C) for the sloped groups. Note that in slow baseline walking (Figure 4C; 1st column) subjects shifted their ankle position backward in the incline condition (i.e., |*X*| > |α|) relative to the body (Figure 4C and green symbols in Figure 4D). The opposite effect (i.e., |*X*| < |α|) was observed for the decline group (Figure 4C and red symbols in Figure 4D). Leg orientation was additionally regulated by walking speed, as shown by the distinct leg orientations between slow (Figure 4C; 1st column) and fast baseline walking (Figure 4C; 2nd column). The demands on each leg to walk at the specific speed and inclination persisted during split-belt walking. Consistently, subjects recovered the speed-specific baseline leg orientation during late adaptation (Figure 4C; 3rd column: black lines with colored squares indicate leg orientations at the speed-specific baselines). The similarity between leg orientations across the baseline and late adaptation epochs in all sloped conditions was quantified by the regression shown in Figure 4D (*y* = 0.93^∗^*x*; *R*^2^ = 0.98, *p* < 0.001). The idealized case, in which baseline and late adaptation values are exactly the same, is also displayed in Figure 4D as a reference (dashed gray line). These leg orientations consequently led to step lengths that were only the same at the end of adaptation in the flat group, but not in the two sloped conditions (e.g., step length in Figure 2, 4C). Lastly, the leading legs’ orientation (α_slow_ or α_fast_) were similar before and after removal of the split perturbation, whereas the trailing legs’ orientation (*X*_slow_ or *X*_fast_) were swapped between legs. In other words, the slow leg’s trailing orientation during post-adaptation (*X*_slow_ in Figure 4E) was similar to the fast leg’s trailing orientation (*X*_fast_) during late adaptation (dotted red line in Figure 4E) and vice versa for the other leg. This switching of the trailing legs’ orientation contributed to the reduced step lengths of the slow leg during post-adaptation (Figure 4E; blue solid line), which was also presented in Figure 3. The ipsilateral similarity between α and the contralateral relationship between *X* from late adaptation to early post-adaptation in all sloped conditions was quantified by the regression in Figure 4F (*y* = 0.99^∗^*x*; *R*^2^ = 0.96, *p* < 0.001). Of note, neither the leading nor the trailing legs’ orientation in post-adaptation matched the respective leg orientations at medium baseline walking (Figure 4E; black lines with black squares), which is the speed at which subjects walked during post-adaptation. Thus, leg orientation exhibited after-effects. In sum, all groups recovered their baseline leg orientation at the expense of step length symmetry.

### Braking and Propulsion Forces Were Predominantly Changed in the Incline and Decline Groups, Respectively

**FIGURE 5:**
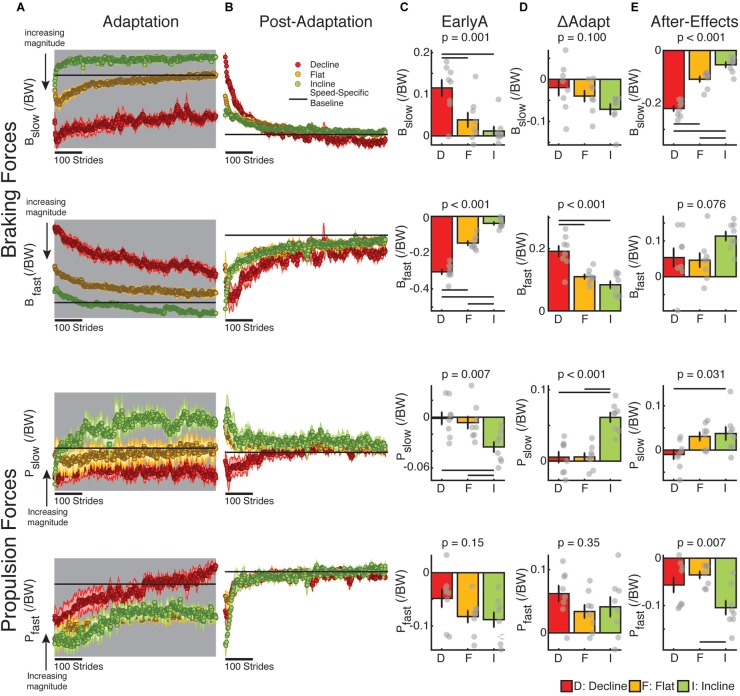
Adaptation and after-effects of braking and propulsion forces. **(A,B)** Stride-by-stride time courses of braking and propulsion forces for each leg are shown during adaptation **(A)**, and post-adaptation **(B)**. Each data point represents the average of 5 consecutive strides and shaded regions indicate the standard error for each group. For visualization purposes, the speed-specific baseline behavior (represented by the black line) was removed for all groups (i.e., the fast baseline for the fast leg during adaptation, the slow baseline for the slow leg during adaptation, and the medium baseline for both legs during post-adaptation). Note that the direction of change in magnitudes are noted as they are different for braking and propulsion forces (e.g., ‘downward’ changes represent an increase in force magnitude for the braking forces, but a decrease in force magnitude for the propulsion forces). **(C–E)** Positive and negative values, respectively, indicate increments or reduction in force relative to speed-specific baselines. Thin horizontal lines between groups illustrate significant differences (*p* < 0.05) based on *post hoc* analysis and gray dots indicate values for individual subjects. **(C)** EarlyA: The height of the bars indicates group averages for braking and propulsion forces for each leg during early adaptation ± standard errors. We observed that the braking and propulsion forces were more perturbed in the decline and incline groups, respectively, compared to the flat group. **(D)** ΔAdapt: The height of the bars indicates group averages for the adaptation of braking and propulsion forces for each leg during split-belt walking ± standard errors. We observed that the adaptation of fast leg braking forces was significantly greater in the decline than the flat group, whereas the adaptation of slow leg propulsion forces was significantly larger in the incline than the flat group. **(E)** After-Effects: The height of the bars indicates group average after-effects during early post-adaptation relative to medium baseline ± standard errors. The preferential impact of decline walking on braking forces and incline walking on propulsion forces was also observed in the after-effects. Namely, the flat group had smaller slow braking force after-effects than the decline group and smaller fast propulsion force after-effects than the incline group.

We assessed the adaptation of forces under the distinct inclination conditions before investigating the relation between kinetic and kinematic measures during and after split-belt walking. We found that braking and propulsion forces were predominantly modified during adaptation and post-adaptation in the sloped condition that naturally prioritized them (Figure 5A,B). In other words, braking was mostly modulated in the decline group, whereas propulsion was primarily modulated in the incline group. This preferential regulation of braking and propulsion forces was indicated by the significant group effect during early adaptation (Figure 5C) for braking forces on both legs (*B*_slow_ EarlyA: *p* = 0.001; *B*_fast_ EarlyA: *p* < 0.001) and propulsion forces of the slow leg (*P*_slow_ EarlyA: *p* = 0.007) but not the fast leg (*P*_fast_ EarlyA: *p* = 0.15). More specifically, the braking and propulsion forces were, respectively, more perturbed in the decline and incline groups compared to the flat group (*B*_slow_ EarlyA: decline vs. flat *p* = 0.014; *B*_fast_ EarlyA: decline vs. flat *p* < 0.001; *P*_slow_ EarlyA: incline vs. flat *p* = 0.028). Despite the group differences during early adaptation, only the forces associated with the step lengths of the fast leg (i.e., fast braking and slow propulsion) were adapted differently across groups (Figure 5D; *B*_fast_ ΔAdapt: *p* < 0.001; *P*_slow_ ΔAdapt: *p* < 0.001), whereas the other forces were adapted similarly across sloped conditions (*B*_slow_ ΔAdapt: *p* = 0.10; *P*_fast_ ΔAdapt: *p* = 0.35). Once again, fast braking was preferentially adapted in the decline group compared to the flat group (*p* = 0.001) and slow propulsion was preferentially adapted in the incline group compared to the flat group (*p* < 0.001). Group analyses on early post-adaptation (Figure 5E) indicated that sloped condition had a significant impact on the after-effects of slow braking (*p* < 0.001), slow propulsion (*p* = 0.031), and fast propulsion (*p* = 0.007) and a trending effect on fast braking (*p* = 0.076). However, only the forces contributing to after-effects of the slow step length (i.e., slow braking and fast propulsion) exhibited greater after-effects than those regularly observed after flat split-belt (*B*_slow_ ΔPost: decline vs. flat *p* < 0.001; *P*_fast_ ΔPost: incline vs. flat *p* = 0.005 in contrast with *P*_slow_ ΔPost: incline vs. flat *p* = 0.98). In sum, inclination conditions predominantly modified the perturbation, adaptation, and after-effects of braking and propulsion forces in a preferential manner: incline walking mostly modified the adaptation of propulsion forces, whereas decline walking mostly altered the adaptation of braking forces.

### Propulsion Forces Predict Subject-Specific Step Length Asymmetry After-Effects

**FIGURE 6:**
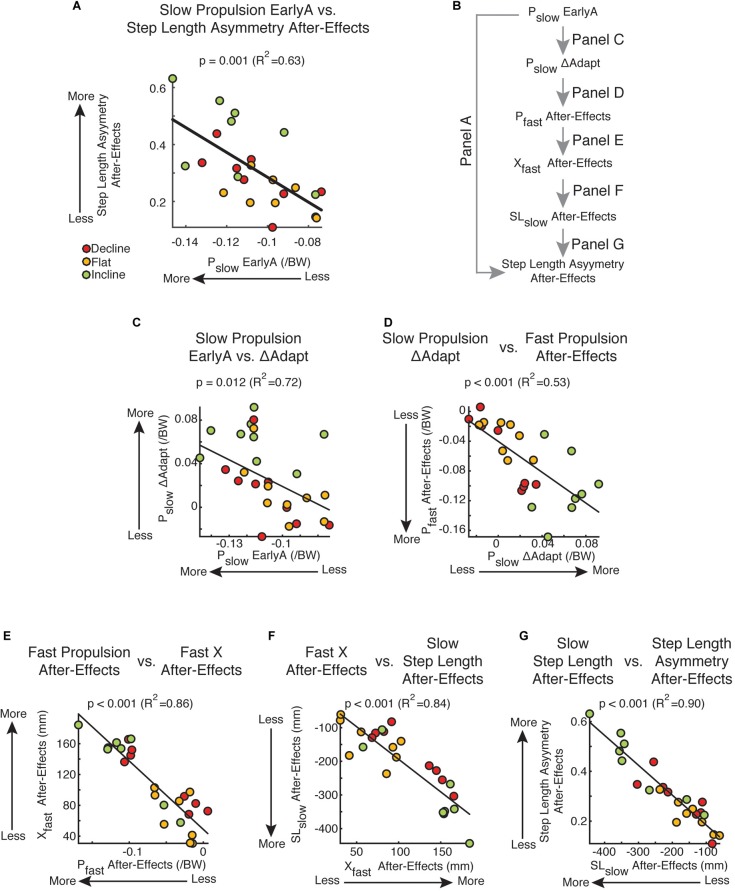
Propulsion-based statistical model and kinetic vs. kinematic regressions. **(A)** Scatter plot of the slow leg’s propulsion force during early adaptation vs. step length asymmetry after-effects. This was the only leg and force component of the AP forces that was significantly related to after-effects out of the 4 force components (*P*_slow_: *p* = 0.001, *R*^2^ = 0.63; data not shown: *P*_fast_: *p* = 0.74, *B*_slow_: *p* = 0.49, *B*_fast_: *p* = 0.49). **(B)** This schematic outlines the correlations presented in **(C–G)** linking the subject-specific propulsion forces during early adaptation (*P*_slow_ EarlyA) and step length asymmetry after-effects. **(C–G)** Results from multiple or univariate linear regressions are indicated. Colored dots illustrate individual values. **(C)** *P*_slow_ EarlyA vs. *P*_slow_ ΔAdapt: Multiple regression indicated that the adaptation of the slow leg’s propulsion force (*P*_slow_ ΔAdapt) depended on the inclination condition and was positively associated to the slow leg’s propulsion force during early adaptation (*P*_slow_ EarlyA). **(D)** *P*_slow_ ΔAdapt vs. *P*_fast_ After-Effects: Multiple regression indicated that the fast leg’s after-effects in propulsion forces depended on the inclination condition and the slow leg’s adaptation of propulsion forces. This contralateral relation between adaptation and post-adaptation is also observed in individual step lengths. **(E)** *P*_fast_ After-Effects vs. *X*_fast_ After-Effects: A positive correlation was found between after-effects in the fast legs’ propulsion force (*P*_fast_ After-Effects) and fast leg’s trailing position (*X*_fast_ After-Effects). **(F)** *X*_fast_ After-Effects vs. SL_slow_ After-Effect: Multiple regression indicated that the after-effects in the slow leg’s step length depended on the inclination condition and was positively associated to the after-effects in the fast trailing leg’s position. **(G)** SL_slow_ After-Effect vs. Step Length Asymmetry After-Effects: A positive correlation was found between after-effects in the slow leg’s step length and step length asymmetry. A similar relation was not found between the fast leg’s step length and step length asymmetry (*p* = 0.46). Thus, step length asymmetries during post-adaptation are mostly attributed to slow step length after-effects.

While braking and propulsion forces were both altered with inclination during and after split-belt walking, only disruptions to the slow leg’s propulsion force during adaptation was indicative of step length asymmetry after-effects during post-adaptation. Namely, only the slow propulsion force during early adaptation was associated to step length asymmetry after-effects (Figure 6A: *P*_slow_: *p* = 0.001, *R*^2^ = 0.63; data not shown: *P*_fast_: *p* = 0.74, *B*_slow_: *p* = 0.49, *B*_fast_: *p* = 0.49). This relation can be explained by the impact of the slow leg’s propulsion force on the slow step length’s after-effects (results summarized in Figure 6B). Specifically, large perturbations of the slow leg’s propulsion force (*P*_slow_ EarlyA) led to large adaptation of this parameter (*P*_slow_ ΔAdapt) (Figure 6C; *p* = 0.012, *R*^2^ = 0.72). In addition, the adaptation of the slow leg’s propulsion force (*P*_slow_ ΔAdapt) was positively associated to After-Effects on the fast leg’s propulsion force (*P*_fast_ After-Effects) (Figure 6D; *p* < 0.001, *R*^2^ = 0.53). This is consistent with the results in Figure 3 indicating that adaptation of step length on one side led to after-effects on the other side. Importantly, there was a strong association between the fast leg’s propulsion after-effects (*P*_fast_ After-Effects) and those of the fast leg’s trailing orientation (*X*_fast_ After-Effects) (Figure 6E; *p* < 0.001, *R*^2^ = 0.86), which have a direct impact on the after-effects of the slow leg’s step length (SL_slow_ After-Effects) (Figure 6F; *p* < 0.001, *R*^2^ = 0.84). Lastly, the after-effects on the slow leg’s step length (SL_slow_ After-Effects), and not the fast one (data not shown), were the ones driving the after-effects in step length asymmetry (Figure 6G; *p* < 0.001, *R*^2^ = 0.90). It is worth pointing out that group was a factor in all these regressions since it was either a significant categorical predictor in the multiple regressions (Figure 6A: *p*_group_ = 0.012; Figure 6C: *p*_group_ < 0.001; Figure 6F: *p*_group_ = 0.015) or significantly correlated to the continuous variables (Figure 6D: Pearson *p* < 0.001; Figure 6E: Pearson *p* = 0.043; Figure 6G: Pearson *p* = 0.024). Thus, these associations depend on the large inter-subject variability facilitated by the different sloped conditions. Taken together, the after-effects in step length asymmetry were unilaterally due to after-effects in the slow leg’s step length, which were facilitated by the large perturbation and adaptation of the slow leg’s propulsion force.

## Discussion

We investigated the influence of anterior–posterior forces on gait adaptation and after-effects induced by split-belt walking at different slopes, which naturally altered leg orientation and forces when feet were in contact with the ground. To our surprise, each inclination group recovered their baseline leg orientation at the expense of step length symmetry, which was a profound finding given that step length asymmetry is considered a biomarker of inefficient gait (Finley et al., 2013; Bhounsule et al., 2014; Awad et al., 2015; Finley and Bastian, 2017). These distinct leg orientations were likely self-selected to generate the forces for walking at the specific speed and slope set by each split-belt task. This was achieved by distinct adaptation of the leading and trailing legs’ orientations at foot landing, suggesting the involvement of different physiological mechanisms in the control of leg orientation over the course of the stance phase. It was also unexpected that propulsion, rather than braking forces, augmented the recalibration of gait. This was indicated by the larger adaptation and after-effects of the sloped group with larger propulsion demands compared to the one with larger braking demands. The key role of propulsion forces was further supported by the fact that perturbation of the propulsion force during early adaptation was predictive of individual after-effects. Taken together, our findings demonstrate that altering propulsion forces during split-belt walking facilitates locomotor adaptation and after-effects. These results suggest that increasing propulsion demands could potentially lead to more gait changes in clinical populations through error-based protocols like split-belt walking.

### The Motor System Prioritizes the Control of Leg Orientation Over Step Length Symmetry During Split-Belt Walking

All groups recovered their baseline leg orientation at the expense of step length symmetry, which indicated that speed-specific leg orientation was prioritized over symmetric step lengths. This has two key implications. First, kinetic demands strongly shape our movements. Notably, inverted pendulum models of walking suggest that subjects orient their legs to maintain a constant speed by equalizing positive and negative work over the gait cycle (Kajita et al., 2001; Donelan et al., 2002; Kuo, 2002; Kuo et al., 2005). Consistently, we observed that subjects oriented their legs in the incline and decline groups to generate the forces counteracting the distinct effect of gravity on the center of mass at the different slopes (Lay et al., 2006). Thus, we believe that subjects in each sloped condition were equally proficient at adapting their movements, but they reached different step lengths during late adaptation because they had distinct kinetic demands, rather than because subjects in the decline or incline group were worse at recovering symmetric steps than those in the flat group. In sum, leg orientation is closely regulated in order to walk at the distinct speeds and inclination (Leroux et al., 2002; Orendurff et al., 2008; Dewolf et al., 2018) imposed on each group. We particularly observed a strong effect of slope on leg orientation when walking slow, but this is also observed at fast speeds when using a coordinate frame aligned with gravity (Leroux et al., 2002), rather than aligned with walking surface as done in this study. It is worth pointing out that there are other factors in addition to leg orientation that are modulated to meet kinetic demands at each slope, such as muscle coordination (Wall-Scheffler et al., 2010), body posture (Leroux et al., 2002), and joint angles (Hsiao et al., 2016). Therefore, although we focused our analysis on leg orientation to explain the asymmetric step lengths across groups, there are other factors that could also influence the differences in locomotor adaptation that we observed across sloped conditions.

A second implication from our results is that step length symmetry is a gait feature that may not be as valued by the motor system as previously considered. Specifically, step length symmetry is thought to be tightly controlled because subjects self-select symmetric step lengths even in asymmetric environments (e.g., Reisman et al., 2005; Savin et al., 2014) and those with more symmetric step lengths in these environments are also those exerting less metabolic energy (Finley et al., 2013). Thus, it was unexpected that subjects in the incline and decline split-belt groups overshot or undershot step length symmetry to recover baseline leg orientations. Given that humans have least-effort tendencies (Margaria, 1976; Alexander, 1989; Bertram and Ruina, 2001; Bertram, 2005; Kuo et al., 2005; Selinger et al., 2015) we think that step length symmetry is not necessarily energetically optimal when walking in sloped surfaces, and perhaps even in flat split-belt environments (Sánchez et al., 2017; Leech et al., 2018). Instead, subjects might self-select leg orientations to optimally generate the mechanical work (Selgrade et al., 2017) for walking at the speed and inclination imposed on each leg, but further studies are needed to determine if this is the case.

The self-selected leg orientation also led to a contralateral relation between adaptation and post-adaptation. In other words, the disruption to forces and movements of individual legs led to more ipsilateral adaptation, but it did not result in larger ipsilateral after-effects. In fact, more adaptation of one leg changed the gait of the other leg as indicated by after-effects in kinetic and kinematic measures (e.g., propulsion forces, step length, and trailing leg orientation). This is in contrast to sensorimotor adaptation of other motor behaviors such as reaching, in which adaptation and de-adaptation effects are mostly constrained to a single effector (Nozaki et al., 2006; Yokoi et al., 2011, 2017; Wang et al., 2013). The contralateral relation between adaptation and post-adaptation might be exclusive to locomotion; possibly because, unlike bimanual tasks (Ahmed et al., 2008; Yokoi et al., 2014), legs share a common goal in walking (i.e., keep the body on the treadmill) while experiencing opposite perturbations (i.e., one leg moves fast and the other leg moves slow) (Choi and Bastian, 2007). This contralateral relation between legs and unilateral effects reported on each group are, of course, occluded when using symmetry measures to characterize locomotor adaptation. Consequently, it is important to characterize each leg’s adjustments in tasks inducing locomotor adaptation (e.g., Reisman et al., 2005), particularly when targeting unilateral deficits on hemiparetic gait (Bowden et al., 2006; Balasubramanian et al., 2007). Another consistent observation in the analysis of individual limbs was that all groups converged to the same step length value for the leg walking slow during adaptation. This is exclusively observed during split and not tied conditions, indicating that this might be a task-constraint of split-belt walking, which will be the subject of future work. In sum, split-belt walking at different slopes predominantly altered the adaptation of one leg and led to subsequent after-effects on the other leg, highlighting the bilateral nature of sensorimotor adaptation in walking.

### Physiological Mechanisms for Regulating Leg Orientation

Our results suggest distinct control between the leading and trailing legs’ orientation when taking a step because they transition differently upon sudden changes in the walking environment. More specifically, the leading leg’s orientation at foot landing (α) exhibits smooth and continuous changes when transitioning from the split to tied situations in all our inclination conditions and other perturbation magnitudes (Malone et al., 2012), whereas the trailing leg’s orientation (*X*) is discontinuous. This suggests that the leading leg’s orientation at foot landing is controlled in a feedforward manner –it is planned before the movement is executed based on a slowly updated internal representation of the environment. This is supported by the fact that sensory information about leg orientation at foot landing is sent to cerebellar structures (Bosco and Poppele, 2001), housing the feedforward control of movements (Herzfeld et al., 2015) and the fact that spinalized cats cannot adapt the orientation of the leading leg during split-belt walking (Frigon et al., 2017). On the other hand, the trailing leg’s orientation when taking a step could be determined by a combination of feedback and feedforward control. Consider that feedback mechanisms adjust our movements by transforming delayed sensory information into actions in real-time (Jordan and Rumelhart, 1992; Bhushan and Shadmehr, 1999). Accordingly, the trailing leg’s orientation is immediately regulated upon manipulations to ipsilateral sensory information from spindles in hip muscles, load sensors, and cutaneous information (Grillner and Rossignol, 1978; Duysens and Pearson, 1980; Duysens et al., 2000; Pang and Yang, 2000; Rossignol, 2006). In addition, the trailing leg’s orientation is determined by the step time (i.e., period between ipsilateral and contralateral heel-strikes) (Finley et al., 2015), which is controlled in a feedforward manner as suggested by behavioral split-belt studies (Malone et al., 2012; Finley et al., 2015) and Purkinjie cells tracking heel-strikes (Apps et al., 1995; Bosco and Poppele, 2001). In particular, the feedforward control of step timing and hence that of the trailing leg’s orientation might be based on subjects’ expectation of the speed at which the belt is moving. In sum, our results suggest distinct control mechanisms of the leading and trailing legs’ orientation when taking a step: the leading position is mostly mediated by feedforward mechanisms, whereas the trailing position is mediated by a combination of feedforward and feedback mechanisms.

### Sensorimotor Recalibration in Walking Increases When Manipulating Propulsion Forces, Rather Than Braking Ones

While braking and propulsion forces were adapted in all groups, augmenting propulsion forces facilitated motor adaptation. This was indicated by the greater adaptation and after-effects of the incline group (larger propulsion demands), whereas the kinematic adaptation was reduced and the after-effects were unchanged in the decline group (larger braking demands). The preferential impact of propulsion on locomotor adaptation was unexpected given prior studies reporting minimal (Roemmich et al., 2012) or absent adaptation of propulsion forces (Ogawa et al., 2014) and subsequently lacking propulsion after-effects (Roemmich et al., 2012; Ogawa et al., 2014) in contrast with the adaptation and after-effects of braking forces (Ogawa et al., 2014). Our findings are at odds with these reports possibly because our participants experienced greater speed differences and were adapted at a more naturalistic walking speed (i.e., mean speed across legs of 1 m/s vs. 0.75 m/s). We additionally found that the incline group adapted faster, indicating that incline walking also augmented the saliency (i.e., easier to detect) and/or sensitivity (i.e., quicker to respond) to the split-belt perturbation. The saliency of the speed difference between legs may be augmented in the incline condition because of reduced cadence (Kawamura et al., 1991; Sun et al., 1996; McIntosh et al., 2006; Phan et al., 2013) and hence longer stance times (i.e., period in direct contact with the environment), which increase subjects’ ability to perceive speed differences (Hoogkamer et al., 2015). In addition, sensory inputs encoding walking speed are stimulated more when walking incline (Sinkjaer et al., 2000; Lamont and Zehr, 2006; Klint et al., 2008; Tillakaratne et al., 2014; Choi et al., 2016) also increasing the saliency of speed differences between the legs in the split situation. On the other hand, subjects in the incline group might have adapted faster because they were more sensitive to increments in energetic cost due to step length asymmetry (Finley et al., 2013; Bhounsule et al., 2014), because incline walking has large energetic demands in and of itself (Johnson et al., 2002). Therefore, larger propulsion demands experienced by the incline group resulted in more and faster gait changes, indicating that altering propulsion forces during split-belt walking facilitated locomotor adaptation.

The relevance of propulsion forces was also evident by the association between individual propulsion forces during early adaptation and subject-specific after-effects. The positive relation between large movement disruptions in early adaptation and after-effects is well documented in error-based learning (Körding and Wolpert, 2004; Wei and Körding, 2009; Green et al., 2010). However, the lack of correlations between the braking force or the fast leg propulsion force strongly suggest that slow leg propulsion forces during adaptation regulate after-effects. Of note, we observed a large range of propulsion forces during early adaptation across individuals, which might be needed to identify the reported association between propulsion forces and after-effects. In sum, our findings indicate that augmenting propulsion demands during split-belt walking facilitates gait adaptation during and after split-belt walking, suggesting that altering propulsion forces could be used as a training stimulus for gait rehabilitation.

### Clinical Implications

Our results might have an impact on the rehabilitation of hemiparetic gait because error-augmentation protocols, like the one presented here, can induce gait improvements in stroke survivors (Reisman et al., 2007; Savin et al., 2014; Reissman et al., 2018) that persist with repeated exposure (Reisman et al., 2013; Lewek et al., 2018). Moreover, we show that increasing propulsion demands during split-belt walking facilitates the adaptation of gait in healthy individuals, as we previously observed in stroke survivors (Sombric et al., 2015). Thus, incline split-belt walking might be beneficial for gait rehabilitation post-stroke because it will overcome the limited gait adaptation of stroke individuals during regular split-belt walking (Malone and Bastian, 2014). In addition, it will target paretic propulsion forces, which directly contribute to gait asymmetry (Bowden et al., 2006; Balasubramanian et al., 2007) and can be modulated by stroke individuals when required by the task (Kesar et al., 2011, 2014; Reisman et al., 2013; Awad et al., 2014; Hsiao et al., 2015, 2016). Of note, previous research indicates that overground walking post-stroke is most improved following decline, rather than incline, interventions due to greater similarity between the flat and decline environments (Carda et al., 2013). Thus, the augmented adaptation in the incline environment may not translate to overground walking. Therefore, the efficacy of our protocol as an intervention will depend on future work assessing its generalization to level walking. Lastly, we find that incline split-belt walking leads to faster adaptation. This could be exploited to increase the slower adaptation rate of older populations (Sombric et al., 2017) who will be the primary recipients of gait rehabilitation. In sum, interventions that increase propulsion demands may be a viable rehabilitation strategy, but future work is necessary to determine the efficacy of incline split-belt walking over other forms of training.

## Author Contributions

GT-O and CS were involved with the conception and design of the work. CS and JC collected and analyzed the data. CS and GT-O interpreted the results. CS drafted the manuscript, which was carefully revised by all authors. The final version of the manuscript has been approved by all the authors who agree to be accountable for all aspects of the work in ensuring that questions related to the accuracy or integrity of any part of the work are appropriately investigated and resolved. All authors qualify for authorship and all those who qualify for authorship are listed.

## Conflict of Interest Statement

The authors declare that the research was conducted in the absence of any commercial or financial relationships that could be construed as a potential conflict of interest.

## References

[B1] Ahmed A. A. Wolpert D. M. Flanagan J. R. (2008). Flexible representations of dynamics are used in object manipulation. *Curr. Biol.* 18 763–768. 10.1016/j.cub.2008.04.061 18485709 PMC2387196

[B2] Alexander R. M. (1989). Optimization and gaits in the locomotion of vertebrates. *Physiol. Rev.* 69 1199–1227. 10.1152/physrev.1989.69.4.1199 2678167

[B3] Apps R. Hartell N. A. Armstrong D. M. (1995). Step phase-related excitability changes in spino- olivocerebellar paths to the c1 and C3 zones in cat cerebellum. *J. Physiol.* 483 (Pt 3), 687–702. 10.1113/jphysiol.1995.sp020614 7776251 PMC1157810

[B4] Awad L. N. Palmer J. A. Pohlig R. T. Binder-Macleod S. A. Reisman D. S. (2015). Walking speed and step length asymmetry modify the energy cost of walking after stroke. *Neurorehabil. Neural Repair* 29 416–423. 10.1177/1545968314552528 25288581 PMC4385745

[B5] Awad L. N. Reisman D. S. Kesar T. M. Binder-Macleod S. A. (2014). Targeting paretic propulsion to improve poststroke walking function: a preliminary study. *Arch. Phys. Med. Rehabil.* 95 840–848. 10.1016/j.apmr.2013.12.012 24378803 PMC4160043

[B6] Balasubramanian C. K. Bowden M. G. Neptune R. R. Kautz S. A. (2007). Relationship between step length asymmetry and walking performance in subjects with chronic hemiparesis. *Arch. Phys. Med. Rehabil.* 88 43–49. 10.1016/j.apmr.2006.10.004 17207674

[B7] Bertram J. E. A. (2005). Constrained optimization in human walking: cost minimization and gait plasticity. *J. Exp. Biol.* 208 979–991. 10.1242/jeb.01498 15767300

[B8] Bertram J. E. A. Ruina A. (2001). Multiple walking speed-frequency relations are predicted by constrained optimization. *J. Theor. Biol.* 209 445–453. 10.1006/jtbi.2001.2279 11319893

[B9] Betschart M. McFadyen B. J. Nadeau S. (2018). Repeated split-belt treadmill walking improved gait ability in individuals with chronic stroke: a pilot study. *Physiother. Theory Pract.* 34 81–90. 10.1080/09593985.2017.1375055 28901824

[B10] Bhounsule P. A. Cortell J. Grewal A. Hendriksen B. Karssen J. G. D. Paul C. (2014). Low-bandwidth reflex-based control for lower power walking: 65 km on a single battery charge. *Int. J. Rob. Res.* 33 1305–1321. 10.1177/0278364914527485

[B11] Bhushan N. Shadmehr R. (1999). Evidence for a forward dynamics model in human adaptive motor control. *Adv. Neural Inf. Process. Syst.* 11 3–9. 14610628

[B12] Bosco G. Poppele R. E. (2001). Proprioception from a spinocerebellar perspective. *Physiol. Rev.* 81 539–568. 10.1152/physrev.2001.81.2.539 11274339

[B13] Bowden M. G. Balasubramanian C. K. Neptune R. R. Kautz S. A. (2006). Anterior-posterior ground reaction forces as a measure of paretic leg contribution in hemiparetic walking. *Stroke* 37 872–876. 10.1161/01.STR.0000204063.75779.8d 16456121

[B14] Carda S. Invernizzi M. Baricich A. Cognolato G. Cisari C. (2013). Does altering inclination alter effectiveness of treadmill training for gait impairment after stroke? A randomized controlled trial. *Clin. Rehabil.* 27 932–938. 10.1177/0269215513485592 23798746

[B15] Choi J. T. Bastian A. J. (2007). Adaptation reveals independent control networks for human walking. *Nat. Neurosci.* 10 1055–1062. 10.1038/nn1930 17603479

[B16] Choi J. T. Jensen P. Nielsen J. B. Bouyer L. J. (2016). Error signals driving locomotor adaptation: cutaneous feedback from the foot is used to adapt movement during perturbed walking. *J. Physiol.* 19 5673–5684. 10.1113/JP271996 27218896 PMC5043025

[B17] Dewolf A. H. Ivanenko Y. Zelik K. E. Lacquaniti F. Willems P. A. (2018). Kinematic patterns while walking on a slope at different speeds. *J. Appl. Physiol.* 125 642–653. 10.1152/japplphysiol.01020.2017 29698109 PMC6842866

[B18] Dewolf A. H. Ivanenko Y. P. Lacquaniti F. Willems P. A. (2017). Pendular energy transduction within the step during human walking on slopes at different speeds. *PLoS One* 12:e0186963. 10.1371/journal.pone.0186963 29073208 PMC5658120

[B19] Donelan J. M. Kram R. Kuo A. D. (2002). Simultaneous positive and negative external mechanical work in human walking. *J. Biomech.* 35 117–124. 10.1016/S0021-9290(01)00169-5 11747890

[B20] Duysens J. Clarac F. Cruse H. Fysica M. National C. Recherche D. (2000). Load-regulating mechanisms in gait and posture: comparative aspects. *Physiol. Rev.* 80 83–133. 10.1152/physrev.2000.80.1.83 10617766

[B21] Duysens J. Pearson K. (1980). Inhibition of flexor burst generation by loading ankle extensor muscles in walking cats. *Brain Res.* 187 321–332. 10.1016/0006-8993(80)90206-1 7370733

[B22] Finley J. M. Bastian A. J. (2017). Associations between foot placement asymmetries and metabolic cost of transport in hemiparetic gait. *Neurorehabil. Neural Repair* 31 168–177. 10.1177/1545968316675428 27798378 PMC5243179

[B23] Finley J. M. Bastian A. J. Gottschall J. S. (2013). Learning to be economical: the energy cost of walking tracks motor adaptation. *J. Physiol.* 591 1081–1095. 10.1113/jphysiol.2012.245506 23247109 PMC3591716

[B24] Finley J. M. Long A. Bastian A. J. Torres-Oviedo G. (2015). Spatial and temporal control contribute to step length asymmetry during split-belt adaptation and hemiparetic gait. *Neurorehabil. Neural. Repair* 29 786–795. 10.1177/1545968314567149 25589580 PMC4501921

[B25] Frigon A. Thibaudier Y. Hurteau M. Dambreville C. (2017). Left–right coordination from simple to extreme conditions during split-belt locomotion in the chronic spinal adult cat. *J. Physiol.* 595 341–361. 10.1113/JP272740 27426732 PMC5199742

[B26] Green D. A. Bunday K. L. Bowen J. Carter T. Bronstein A. M. (2010). What does autonomic arousal tell us about locomotor learning? *Neuroscience* 170 42–53. 10.1016/j.neuroscience.2010.06.079 20620200

[B27] Grillner S. Rossignol S. (1978). On the initiation of the swing phase of locomotion in chronic spinal cats. *Brain Res.* 146 269–277. 10.1016/0006-8993(78)90973-3 274169

[B28] Herzfeld D. J. Kojima Y. Soetedjo R. Shadmehr R. (2015). Encoding of action by the Purkinje cells of the cerebellum. *Nature* 526 439–441. 10.1038/nature15693 26469054 PMC4859153

[B29] Hoogkamer W. Bruijn S. M. Potocanac Z. Van Calenbergh F. Swinnen S. P. Duysens J. (2015). Gait asymmetry during early split-belt walking is related to perception of belt speed difference. *J. Neurophysiol.* 114 1705–1712. 10.1152/jn.00937.2014 26203114 PMC4567612

[B30] Hsiao H. Knarr B. A. Higginson J. S. Binder-Macleod S. A. (2015). Mechanisms to increase propulsive force for individuals poststroke. *J. Neuroeng. Rehabil.* 12 1–8. 10.1186/s12984-015-0030-8 25898145 PMC4406180

[B31] Hsiao H. Y. Zabielski T. M. Palmer J. A. Higginson J. S. Binder-Macleod S. A. (2016). Evaluation of measurements of propulsion used to reflect changes in walking speed in individuals poststroke. *J. Biomech.* 49 4107–4112. 10.1016/j.jbiomech.2016.10.003 27756571 PMC5164961

[B32] Item-glatthorn J. F. Casartelli N. C. Maffiuletti N. A. (2016). Reproducibility of gait parameters at different surface inclinations and speeds using an instrumented treadmill system. *Gait Posture* 44 259–264. 10.1016/j.gaitpost.2015.12.037 27004668

[B33] Johnson A. T. Benhur Benjamin M. Silverman N. (2002). Oxygen consumption, heat production, and muscular efficiency during uphill and downhill walking. *Appl. Ergon.* 33 485–491. 10.1016/S0003-6870(02)00031-5 12236658

[B34] Jordan M. I. Rumelhart D. E. (1992). Forward models: supervised learning with a distal teacher. *Cogn. Sci.* 16 307–354. 10.1207/s15516709cog1603_1

[B35] Jørgensen C. K. Fink P. Olesen F. (2000). Psychological distress among patients with musculoskeletal illness in general practice. *Psychosomatics* 41 321–329. 10.1176/appi.psy.41.4.321 10906354

[B36] Kajita S. Kanehiro F. Kaneko K. Yokoi K. Hirukawa H. (2001). “The 3D linear inverted pendulum mode: a simple modeling for a biped walking pattern generation,” in *Proceedings of the Intelligent Robots and Systems, IIEEE RSJ International Conference*, (Piscataway, NY: IEEE), 239–246. 10.1109/IROS.2001.973365

[B37] Kawamura K. Tokuhiro A. Takechi H. (1991). Gait analysis of slope walking: a study on step length, stride width, time factors and deviation in the center of pressure. *Acta Med. Okayama* 45 179–184. 1891977 10.18926/AMO/32212

[B38] Kesar T. M. Reisman D. S. Perumal R. Jancosko A. M. Higginson J. S. Rudolph K. S. (2011). Combined effects of fast treadmill walking and functional electrical stimulation on post-stroke gait. *Gait Posture* 33 309–313. 10.1016/j.gaitpost.2010.11.019 21183351 PMC3042540

[B39] Kesar T. M. Sauer M. J. Binder-Macleod S. A. Reisman D. S. (2014). Motor learning during poststroke gait rehabilitation: a case study. *J. Neurol. Phys. Ther.* 38 183–189. 10.1097/NPT.0000000000000047 24933501

[B40] Klint R. A. Nielsen J. B. Cole J. Sinkjaer T. Grey M. J. (2008). Within-step modulation of leg muscle activity by afferent feedback in human walking. *J. Physiol.* 586 4643–4648. 10.1113/jphysiol.2008.155002 18669536 PMC2614048

[B41] Körding K. P. Wolpert D. M. (2004). The loss function of sensorimotor learning. *Proc. Natl. Acad. Sci. U.S.A.* 101 9839–9842. 10.1073/pnas.0308394101 15210973 PMC470761

[B42] Kuo A. D. (2002). Energetics of actively powered locomotion using the simplest walking model. *J. Biomech. Eng.* 124:113. 11871597 10.1115/1.1427703

[B43] Kuo A. D. Donelan J. M. Ruina A. (2005). Energetic consequences of walking like an inverted pendulum: step-to-step transitions. *Exerc. Sport Sci. Rev.* 33 88–97. 10.1097/00003677-200504000-00006 15821430

[B44] Lamont E. V. Zehr E. P. (2006). Task-specific modulation of cutaneous reflexes expressed at functionally relevant gait cycle phases during level and incline walking and stair climbing. *Exp. Brain Res.* 173 185–192. 10.1007/s00221-006-0586-4 16821052

[B45] Lay A. N. Hass C. J. Gregor R. J. (2006). The effects of sloped surfaces on locomotion: A kinematic and kinetic analysis. *J. Biomech.* 39 1621–1628. 10.1016/j.jbiomech.2005.05.005 15990102

[B46] Lay A. N. Hass C. J. Nichols T. R. Gregor R. J. (2007). The effects of sloped surfaces on locomotion: an electromyographic analysis. *J. Biomech.* 40 1276–1285. 10.1016/j.jbiomech.2006.05.023 16872616

[B47] Leech K. A. Roemmich R. T. Day K. A. Bastian A. J. (2018). Movement and perception recalibrate differently across multiple days of locomotor learning. *J. Neurophysiol.* 114 608–623. 10.1152/jn.00355.2018 30183471 PMC6230790

[B48] Leroux A. Fung J. Barbeau H. (2002). Postural adaptation to walking on inclined surfaces: I. Normal strategies. *Gait Posture* 15 64–74. 10.1016/S0966-6362(01)00181-3 11809582

[B49] Lewek M. D. Braun C. H. Wutzke C. (2018). The role of movement errors in modifying spatiotemporal gait asymmetry post stroke: a randomized controlled trial. *Clin. Rehabil.* 32 161–172. 10.1177/0269215517723056 28750549 PMC5748372

[B50] Malone L. Bastian A. Torres-Oviedo G. (2012). How does the motor system correct for errors in time and space during locomotor adaptation? *J. Neurophysiol.* 108 672–683. 10.1152/jn.00391.2011 22514294 PMC4073916

[B51] Malone L. A. Bastian A. J. (2014). Spatial and temporal asymmetries in gait predict split-belt adaptation behavior in stroke. *Neurorehabil. Neural Repair* 28 230–240. 10.1177/1545968313505912 24243917 PMC4336782

[B52] Margaria R. (1976). *Biomechanics and Energetics of Muscular Exercise.* Oxford: Clarendon Press.

[B53] Mawase F. Haizler T. Bar-Haim S. Karniel A. (2013). Kinetic adaptation during locomotion on a split-belt treadmill. *J. Neurophysiol.* 109 2216–2227. 10.1152/jn.00938.2012 23365187

[B54] McIntosh A. S. Beatty K. T. Dwan L. N. Vickers D. R. (2006). Gait dynamics on an inclined walkway. *J. Biomech.* 39 2491–2502. 10.1016/j.jbiomech.2005.07.025 16169000

[B55] Nozaki D. Kurtzer I. Scott S. H. (2006). Limited transfer of learning between unimanual and bimanual skills within the same limb. *Nat. Neurosci.* 9 1364–1366. 10.1038/nn1785 17028583

[B56] Ogawa T. Kawashima N. Ogata T. Nakazawa K. (2014). Predictive control of ankle stiffness at heel contact is a key element of locomotor adaptation during split-belt treadmill walking in humans. *J. Neurophysiol.* 111 722–732. 10.1152/jn.00497.2012 24225544

[B57] Orendurff M. S. Bernatz G. C. Schoen J. A. Klute G. K. (2008). Kinetic mechanisms to alter walking speed. *Gait Posture* 27 603–610. 10.1016/j.gaitpost.2007.08.004 17920886

[B58] Pang M. Y. C. Yang J. F. (2000). The initiation of the swing phase in human infant stepping: Importance of hip position and leg loading. *J. Physiol.* 528 389–404. 10.1111/j.1469-7793.2000.00389.x 11034628 PMC2270131

[B59] Patterson K. K. Nadkarni N. K. Black S. E. McIlroy W. E. (2012). Gait symmetry and velocity differ in their relationship to age. *Gait Posture* 35 590–594. 10.1016/j.gaitpost.2011.11.030 22300728 PMC3914537

[B60] Phan P. L. Blennerhassett J. M. Lythgo N. Dite W. Morris M. E. (2013). Over-ground walking on level and sloped surfaces in people with stroke compared to healthy matched adults. *Disabil. Rehabil.* 35 1302–1307. 10.3109/09638288.2012.729646 23210802

[B61] Reisman D. S. Block H. J. Bastian A. J. Darcy S. Block H. J. Bastian A. J. (2005). Interlimb coordination during locomotion: what can be adapted and stored? *J. Neurophysiol.* 94 2403–2415. 10.1152/jn.00089.2005 15958603

[B62] Reisman D. S. McLean H. Keller J. Danks K. A. Bastian A. J. (2013). Repeated split-belt treadmill training improves poststroke step length asymmetry. *Neurorehabil. Neural Repair* 27 460–468. 10.1177/1545968312474118 23392918 PMC3738184

[B63] Reisman D. S. Wityk R. Silver K. Bastian A. J. (2007). Locomotor adaptation on a split-belt treadmill can improve walking symmetry post-stroke. *Brain* 130 1861–1872. 10.1093/brain/awm035 17405765 PMC2977955

[B64] Reisman D. S. Wityk R. Silver K. Bastian A. J. (2009). Split-belt treadmill adaptation transfers to overground walking in persons poststroke. *Neurorehabil. Neural Repair* 23 735–744. 10.1177/1545968309332880 19307434 PMC2811047

[B65] Reissman M. E. Gordon K. E. Dhaher Y. Y. (2018). Manipulating post-stroke gait: Exploiting aberrant kinematics. *J. Biomech.* 67 129–136. 10.1016/j.jbiomech.2017.11.031 29248191

[B66] Roemmich R. T. Stegemöller E. L. Hass C. J. (2012). Lower extremity sagittal joint moment production during split-belt treadmill walking. *J. Biomech.* 45 2817–2821. 10.1016/j.jbiomech.2012.08.036 22985473 PMC3491168

[B67] Rossignol S. (2006). Dynamic sensorimotor interactions in locomotion. *Physiol. Rev.* 86 89–154. 10.1152/physrev.00028.2005 16371596

[B68] Sánchez N. Park S. Finley J. M. (2017). Evidence of energetic optimization during adaptation differs for metabolic, mechanical, and perceptual estimates of energetic cost. *Sci. Rep.* 7:7682. 10.1038/s41598-017-08147-y 28794494 PMC5550492

[B69] Savin D. N. Morton S. M. Whitall J. (2014). Generalization of improved step length symmetry from treadmill to overground walking in persons with stroke and hemiparesis. *Clin. Neurophysiol.* 125 1012–1020. 10.1016/j.clinph.2013.10.044 24286858 PMC3981945

[B70] Selgrade B. P. Thajchayapong M. Lee G. E. Toney M. E. Chang Y.-H. (2017). Changes in mechanical work during neural adaptation to asymmetric locomotion. *J. Exp. Biol.* 220 2993–3000. 10.1242/jeb.149450 28596214 PMC5576064

[B71] Selinger J. C. Shawn M. Connor O. Wong J. D. Maxwell J. Selinger J. C. (2015). Humans can continuously optimize energetic cost during walking report humans can continuously optimize energetic cost during walking. *Curr. Biol.* 25 2452–2456. 10.1016/j.cub.2015.08.016 26365256

[B72] Sinkjaer T. Andersen J. B. Ladouceur M. Christensen L. O. Nielsen J. B. (2000). Major role for sensory feedback in soleus EMG activity in the stance phase of walking in man. *J. Physiol.* 523 817–827. 10.1111/j.1469-7793.2000.00817.x 10718758 PMC2269822

[B73] Sombric C. J. Calvert J. S. Torres-Oviedo G. (2018). Large propulsion demands increase locomotor learning at the expense of step length symmetry. *bioRxiv* [Preprint] 10.1101/372425 PMC6376174 30800072

[B74] Sombric C. J. Harker H. M. Sparto P. J. Torres-oviedo G. (2017). Explicit action switching interferes with the context-specificity of motor memories in older adults. *Front. Aging Neurosci.* 9:40. 10.3389/fnagi.2017.00040 28321188 PMC5337495

[B75] Sombric C. J. Mariscal D. M. Calvert J. S. Iturralde P. A. Torres-Oviedo G. (2015). It’s all uphill from here: incline split-belt walking increases locomotor learning post-stroke. *Adv. Motor Learn. Motor Control.* Available at: https://sites.google.com/site/acmcconference/

[B76] Sun J. M. W. Svensson N. Lloyd D. (1996). The influence of surface slope on human gait characteristics: a study of urban pedestrians walking on an inclined surface. *Ergonomics* 39 677–692. 10.1080/00140139608964489 8854986

[B77] Tillakaratne N. J. K. Duru P. Fujino H. Zhong H. Xiao M. S. Edgerton V. R. (2014). Identification of interneurons activated at different inclines during treadmill locomotion in adult rats. *J. Neurosci. Res.* 92 1714–1722. 10.1002/jnr.23437 24975393

[B78] Wall-Scheffler C. M. Chumanov E. Steudel-Numbers K. Heiderscheit B. (2010). Electromyography activity across gait and incline: the impact of muscular activity on human morphology. *Am. J. Phys. Anthropol.* 143 601–611. 10.1002/ajpa.21356 20623603 PMC3011859

[B79] Wang J. Lei Y. Xiong K. Marek K. (2013). Substantial generalization of sensorimotor learning from bilateral to unilateral movement conditions. *PLoS One* 8:e58495. 10.1371/journal.pone.0058495 23505519 PMC3591418

[B80] Wei K. Körding K. (2009). Relevance of error: what drives motor adaptation? *J. Neurophysiol.* 101 655–664. 10.1152/jn.90545.2008 19019979 PMC2657056

[B81] Yokoi A. Bai W. Diedrichsen J. (2017). Restricted transfer of learning between unimanual and bimanual finger sequences. *J. Neurophysiol.* 117 1043–1051. 10.1152/jn.00387.2016 27974447 PMC5338615

[B82] Yokoi A. Hirashima M. Nozaki D. (2011). Gain field encoding of the kinematics of both arms in the internal model enables flexible bimanual action. *J. Neurosci.* 31 17058–17068. 10.1523/JNEUROSCI.2982-11.2011 22114275 PMC6623869

[B83] Yokoi A. Hirashima M. Nozaki D. (2014). Lateralized sensitivity of motor memories to the kinematics of the opposite arm reveals functional specialization during bimanual actions. *J. Neurosci.* 34 9141–9151. 10.1523/JNEUROSCI.2694-13.2014 24990934 PMC6608246

[B84] Yokoyama H. Sato K. Ogawa T. Yamamoto S.-I. Nakazawa K. Kawashima N. (2018). Characteristics of the gait adaptation process due to split-belt treadmill walking under a wide range of right-left speed ratios in humans. *PLoS One* 13:e0194875. 10.1371/journal.pone.0194875 29694404 PMC5918641

